# Development and Performance of the Pulmonary Embolism Result Forecast Model (PERFORM) for Computed Tomography Clinical Decision Support

**DOI:** 10.1001/jamanetworkopen.2019.8719

**Published:** 2019-08-07

**Authors:** Imon Banerjee, Miji Sofela, Jaden Yang, Jonathan H. Chen, Nigam H. Shah, Robyn Ball, Alvin I. Mushlin, Manisha Desai, Joseph Bledsoe, Timothy Amrhein, Daniel L. Rubin, Roham Zamanian, Matthew P. Lungren

**Affiliations:** 1 Department of Biomedical Data Science, Stanford University, Stanford, California; 2 Department of Radiology, Stanford University, Stanford, California; 3 Duke University Health System, Duke University School of Medicine, Durham, North Carolina; 4 Quantitative Science Unit, Stanford University, Stanford, California; 5 Department of Medicine (Biomedical Informatics), Stanford University, Stanford, California; 6 Department of Medicine, Weill Cornell Medical College, Cornell University, Ithaca, New York; 7 Department of Emergency Medicine, Intermountain Medical Center, Salt Lake City, Utah; 8 Department of Radiology, Duke University School of Medicine, Durham, North Carolina; 9 Department of Medicine, Med/Pulmonary, and Critical Care Medicine, Stanford University, Stanford, California

## Abstract

**Importance:**

Pulmonary embolism (PE) is a life-threatening clinical problem, and computed tomographic imaging is the standard for diagnosis. Clinical decision support rules based on PE risk-scoring models have been developed to compute pretest probability but are underused and tend to underperform in practice, leading to persistent overuse of CT imaging for PE.

**Objective:**

To develop a machine learning model to generate a patient-specific risk score for PE by analyzing longitudinal clinical data as clinical decision support for patients referred for CT imaging for PE.

**Design, Setting, and Participants:**

In this diagnostic study, the proposed workflow for the machine learning model, the Pulmonary Embolism Result Forecast Model (PERFORM), transforms raw electronic medical record (EMR) data into temporal feature vectors and develops a decision analytical model targeted toward adult patients referred for CT imaging for PE. The model was tested on holdout patient EMR data from 2 large, academic medical practices. A total of 3397 annotated CT imaging examinations for PE from 3214 unique patients seen at Stanford University hospitals and clinics were used for training and validation. The models were externally validated on 240 unique patients seen at Duke University Medical Center. The comparison with clinical scoring systems was done on randomly selected 100 outpatient samples from Stanford University hospitals and clinics and 101 outpatient samples from Duke University Medical Center.

**Main Outcomes and Measures:**

Prediction performance of diagnosing acute PE was evaluated using ElasticNet, artificial neural networks, and other machine learning approaches on holdout data sets from both institutions, and performance of models was measured by area under the receiver operating characteristic curve (AUROC).

**Results:**

Of the 3214 patients included in the study, 1704 (53.0%) were women from Stanford University hospitals and clinics; mean (SD) age was 60.53 (19.43) years. The 240 patients from Duke University Medical Center used for validation included 132 women (55.0%); mean (SD) age was 70.2 (14.2) years. In the samples for clinical scoring system comparisons, the 100 outpatients from Stanford University hospitals and clinics included 67 women (67.0%); mean (SD) age was 57.74 (19.87) years, and the 101 patients from Duke University Medical Center included 59 women (58.4%); mean (SD) age was 73.06 (15.3) years. The best-performing model achieved an AUROC performance of predicting a positive PE study of 0.90 (95% CI, 0.87-0.91) on intrainstitutional holdout data with an AUROC of 0.71 (95% CI, 0.69-0.72) on an external data set from Duke University Medical Center; superior AUROC performance and cross-institutional generalization of the model of 0.81 (95% CI, 0.77-0.87) and 0.81 (95% CI, 0.73-0.82), respectively, were noted on holdout outpatient populations from both intrainstitutional and extrainstitutional data.

**Conclusions and Relevance:**

The machine learning model, PERFORM, may consider multitudes of applicable patient-specific risk factors and dependencies to arrive at a PE risk prediction that generalizes to new population distributions. This approach might be used as an automated clinical decision-support tool for patients referred for CT PE imaging to improve CT use.

## Key Points

**Question:**

Can machine-learning approaches achieve an objective pulmonary embolism risk score by analyzing temporal patient data to accurately inform computed tomographic imaging decisions?

**Findings:**

In this multi-institutional diagnostic study of 3214 patients, a machine learning model was designed to achieve an accurate patient-specific risk score for pulmonary embolism diagnosis. The model was successfully evaluated in both multi-institutional inpatient and outpatient settings.

**Meaning:**

Machine learning algorithms using retrospective temporal patient data appear to be a valuable and feasible tool for accurate computation of patient-specific risk score to better inform clinical decision-making for computed tomographic pulmonary embolism imaging.

## Teaser

This diagnostic study evaluates whether a machine learning model, the Pulmonary Embolism Result Forecast Model (PERFORM), can compute a patient-specific risk score for pulmonary embolism as clinical decision support for computed tomography.

## Introduction

Pulmonary embolism (PE) is a common life-threatening clinical problem and computed tomographic (CT) imaging is the standard for diagnosis.^1,2^ The past 20 years have seen an increase in the number of CT imaging examinations performed for PE evaluation and a decrease in imaging yield; approximately 10% or less of patients who undergo CT imaging for PE are positive for the disease^3^ and a recent publication reported positive PE CT yield as less than 1%.^4^ Imaging carries risks due to radiation, intravenous contrast material, and discovery of low incidental imaging findings that further expose patients to unneeded procedures and tests.^5^ It has been estimated that approximately one-third of all CT angiography imaging studies for PE are avoidable and cost the health care system more than $100 million annually.^6^

To improve yield and reduce unneeded CT examinations, systematic use of PE risk scores (ie, Wells or revised Geneva [rGeneva]) is recommended as clinical decision support (CDS), but only a reported minority of clinicians are compliant.^7,8,9^ The deadline in the Protecting Access to Medicare Act will more forcefully mandate consultation with a CDS tool before ordering CT for PE such that eventually studies that are ordered without CDS tools will not be paid.^10^ However, there are limitations with current CDS tools that call their utility into question; a large meta-analysis found no overall improvement in use of CT imaging following CDS implementation based on PE risk scores.^11^ Limitation of the current CDS tools may be explained, in part, by inclusion of subjective criteria and the fact that many known clinical PE risk factors or risk modifiers are not considered in current PE risk scores, which can compel clinicians to order CT imaging against guidelines.^12,13^ Thus, without new scoring systems that can better leverage advances in data science to inform CDS tools, this legislation may lead to widespread adoption of existing PE risk scores and the accompanying well-described limitations.^12,13^

Designing a method that can use routinely collected patient health care data to arrive at patient-specific disease outcome prognosis, if successful, could better inform care decisions for patients with suspected PE. Existing PE risk scores are based on hand-selected clinical variables (eg, expected elevated heart rate and oxygen desaturation as physical manifestations of symptomatic PE), whereas modern machine learning approaches have been shown to handle large volumes of relatively complex interdependent data, are tolerant of errors, and include variables from a variety of sources to provide more personalized estimations with improved accuracy.^14,15,16,17,18^ Estimation of the likelihood of PE may serve as a useful case for application of such powerful tools because (1) the definitive diagnosis can be made on CT imaging, (2) there are numerous factors known to contribute to PE risk, (3) there are many existing rules-based prediction tools against which to compare the performance of a new approach, and (4) overuse of CT for PE diagnosis is of broad concern to clinicians, patients, payers, and regulatory bodies.

The purpose of this study was to design and evaluate a machine learning modeling approach called Pulmonary Embolism Result Forecast Model (PERFORM) for predicting PE imaging outcomes based on patient electronic medical record (EMR) data, including variables such as demographics, vital signs (absolute and change from baseline), diagnoses, medications, and laboratory test results to provide a patient-specific risk score for those referred for CT imaging for PE; we evaluated this model using intrainstitutional and extrainstitutional patient data and compared it with existing PE risk scoring systems.

## Methods

**Figure 1.**
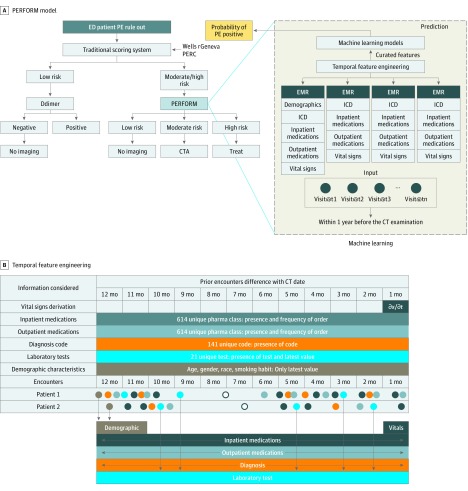
Overview of the Pulmonary Embolism (PE) Prediction Pipeline A, Pulmonary Embolism Result Forecast Model (PERFORM): the proposed workflow begins with all raw structured electronic medical record (EMR) data within 1 year prior to the encounter that is then arranged as a timeline into feature vectors. A machine learning model is then trained with the feature vectors labeled with ground truth PE outcome data to arrive at a model capable of predicting the probability of PE for an unseen patient from the holdout internal and external data set. This can be applied to provide a risk score in clinical decision support for patients referred for computed tomographic (CT) imaging for PE. B, Overview of the temporal feature engineering (each type of EMR data is color coded) and example encounters of 2 patients and how the features have been computed by preserving temporal sequence. CTA indicates CT angiography; ED, emergency department; ICD, *International Classification of Diseases*; and PERC, Pulmonary Embolism Rule-out Criteria.

Figure 1A illustrates the proposed workflow that transforms raw EMR data arranged as a timeline into feature vectors and uses a machine learning model to estimate the PE imaging outcomes to facilities’ clinical decisions. To train the model, we followed the Standards for Reporting of Diagnostic Accuracy (STARD) reporting guideline and used the area under the receiver operating characteristic (AUROC) value to report the performance. Trained models are validated on Stanford University hospital and clinics (SHC) and Duke University Medical Center (Duke) holdout test sets as well as tested on separate SHC and Duke outpatient populations. We compared traditional machine learning models and a new deep learning model against 3 popular clinical scoring systems: Wells, Pulmonary Embolism Rule-out Criteria (PERC), and rGeneva. As a primary measure, the accuracy of the prediction was evaluated by the AUROC curve, which is a well-accepted single-performance measure to show the trade-off between true-positive and false-positive prediction rates. The Duke University Institutional Review Board and Stanford University Institutional Review Board approved the study with waiver of informed consent because of the use of deidentified data.

### Data Used

#### SHC Data Set

With the approval of the Stanford University Institutional Review Board, we retrieved 137 834 contrast-enhanced CT chest examinations performed between January 1, 1998, and January 1, 2016, at SHC, including inpatient, emergency department, and outpatient scans. From the EMRs, we also extracted the clinical data with time stamps and CT imaging report text. To create an annotated data set for training and testing of the PE prediction model, 4512 CT images conducted with PE protocol (PE-CT) examinations were selected from the internal data set of adult patients (after 2008); these data were a stratified random sample (approximately balanced between PE-positive and PE-negative) selected from the study population to capture equal distribution of PE-positive and PE-negative labels for obtaining a balanced data set for training the model. To make a valid comparison with clinical scorings, we created a data set by selecting 100 outpatient consecutive samples from SHC PE-CT data set to generate a separate population for computing pretest probability scores (eFigure 1 in the Supplement).

Each case was manually annotated by 3 experienced radiologists assigned 2 binary class labels (PE present or absent and PE acute or chronic) and the raters were consistent, with κ scores of 0.959 and 0.969, respectively.^19,20^ Because we engineered the model to identify acute PE, we only used examinations labeled as negative for PE (PE-negative) and positive acute (PE-positive) to generate the final annotations for our cohorts. A senior radiologist (M.P.L.) resolved all conflicting findings on the cases manually for preparing the ground truth labels.

#### Duke Data Set

With the approval of the Duke Institutional Review Board, as an external data set, we collected 227 809 contrast-enhanced CT examinations of the chest from Duke performed between January 1, 2013, and August 31, 2017, including inpatient, emergency department, and outpatient scans. We retrieved a set of clinical data of these patients similar to those from SHC with encounter time-stamp details. To create an external annotated data set for validation, we randomly sampled 300 PE-CT examinations from Duke and performed manual annotation with the same group of radiologists. As with SHC, we created a separate set with only consecutive outpatient samples (101 patients) from Duke to compare with clinical scoring systems.

After exclusion of all patients with chronic PE, 3397 annotated PE-CT examinations from 3214 unique patients from SHC were curated as an internal annotated data set, and 244 annotated PE-CT examinations from 240 unique patients from Duke were curated as an external data set. In addition, we created a separate outpatient data set for SHC (100 consecutive patients) and Duke (101 consecutive patients), and these cases are independent from the internal SHC and Duke holdout data set. Cohort assembly is presented as a flowchart in eFigure 1 in the Supplement and characteristics of the patients are presented in Table 1.

**Table 1.** Stratified Patient Characteristics of the Internal (SHC) and External (Duke) Data Set

Variable	No. (%)			
	SHC Inpatients (n = 3214)	Duke Inpatients (n = 240)	SHC Outpatients (n = 100)	Duke Outpatients (n = 101)
Age, mean (SD)	60.53 (19.43)	70.2 (14.2)	57.74 (19.87)	73.06 (15.3)
Sex				
Men	1510 (47.0)	108 (45.0)	33 (33)	42 (41.83)
Women	1704 (53.0)	132 (55.0)	67 (67.0)	59 (58.4)
Race/ethnicity				
White	1952 (60.73)	120 (50.0)	59 (59.0)	56 (55.4)
Black	194 (6.03)	97 (40.4)	11 (11.0)	42 (41.6)
Asian	342 (10.65)	0	6 (6.0)	0
Native American	65 (0.2)	10 (0.4)	5 (5.0)	0
Other	413 (12.83)	10 (0.4)	9 (9.0)	1 (0.01)
Unknown	248 (7.71)	3 (1.3)	10 (10.0)	2 (1.1)
d-Dimer test				
Total d-dimer tests	637 (19.8)	31 (13.11)	29 (29.0)	32 (31.7)
Negative d-dimer test result	42 (6.6)	30 (96.8)	2 (2.0)	4 (4.0)
Prior diseases of pulmonary circulation	495 (15.4)	35 (14.6)	55 (55.0)	13 (12.9)
Cancer	1376 (42.8)	61 (25.4)	34 (34.0)	21 (20.8)
Anticoagulant therapy	1180 (36.7)	53 (22.1)	55 (55.0)	27 (26.53)
Ground truth: acute PE-positive	1967 (61.2)	38 (15.8)	29 (29.0)	23 (22.77)

Abbreviations: Duke, Duke University Medical Center; PE, pulmonary embolism; SHC, Stanford University hospital and clinics.

### Temporal Feature Engineering

For all patients, we defined their observation window as the 12 months leading up to their prediction date (day before CT examination). Within the observation window, we created a feature engineering pipeline that computes a vector representation of the EMR snapshot of each patient by considering the temporal sequence within the records (Figure 1B and the eAppendix in the Supplement provide details). Given the complexity of the EMR data and the requirement of temporality preservation, we designed the pipeline to parse varying types of EMR: (1) all diagnosis codes (except the current encounter); (2) all inpatient and outpatient medications, (3) all raw values of data from laboratory tests, (4) all vital sign data, and (5) all demographic data. The feature engineering method curates the EMR and is tolerant of sparse records (common limitation) (eAppendix, eTable 2, and eTable 3 in the Supplement). We standardized features by removing the mean and scaling to unit variance. Centering and scaling happen independently on each feature by computing the relevant statistics on the samples in the training set. The mean (SD) is then used on later data (ie, internal holdout test and external test set), using the same transformation function.

### Traditional Machine Learning: ElasticNet Model

We created a prediction model based on ElasticNet regularized regression^21^ that combines L1 penalties of lasso and L2 penalties of ridge to overcome the limitations of incorporating high-dimensional features for a relatively small number of samples. We performed shrinkage of the large, quantitative scaled feature matrix while maintaining the pairwise correlation between features and selected a set of discriminative features for distinguishing PE-positive and PE-negative findings (eFigure 3 in the Supplement). The optimal value of the hyperparameters is derived by analyzing the coefficient of the features computed by the 10-fold cross validation on the SHC training data. In addition to ElasticNet, we experimented with other traditional machine learning models (logistic regression, random forest, and adaptive boosting) and summarized the results in the eAppendix in the Supplement.

### Deep Learning: PE Neural Model

We developed an artificial neural network model to automatically classify patient-level PE likelihood in which the first layer of the neural network model takes a temporal feature matrix as input. The output of the first layer then passes through the multiple hidden layers where selecting the number of hidden layers in the model requires balancing the capture of complex data representations against overfitting (variance vs bias) (eFigure 4 in the Supplement). Grid search on 10% validation data selected from the SHC training data was used to identify the hyperparameters, and we experimented with different settings of number of hidden layers, learning rate, activation function, optimizers, number of epochs, and dropout rate. The top 50 performance of the model on the validation data are presented in eTable 5 in the Supplement. The network also contained a dropout layer between the nonlinear hidden layers and output layer with sigmoid activation to prevent overfitting to the training data. The final classification layer of the model contains hidden layers and a sigmoid layer, the purpose of which is to predict the final probability as a differentiable output.

### Statistical Analysis

We chose the AUROC as our primary evaluation measurement. In addition to AUROC, we performed sensitivity analyses to better evaluate the top model’s potential clinical utility. To validate the overfitting and overoptimistic performance claims, which are common concerns for machine learning models, we evaluated the models in 4 different settings.

#### Setting 1: Internal Holdout

We randomly selected 340 CT examinations (10.6%) from the SHC cases for testing and the remaining data were used for training the machine learning models. We verified that none of the patients were duplicated between training and testing. We trained both models on the SHC training data where 10% of the data were used as validation to tune the machine learning hyperparameters: ElasticNet parameters, λ and α; and PE neural models parameters, number of neurons and hidden layers, as well as dropout rate.

#### Setting 2: External Data

We used the models that were trained including only the SHC patients (setting 1) and tested them on 244 annotated CT examinations from Duke, which was curated as an external data set and was not available at the time of prediction model development. This setting allowed us to test models’ reproducibility on independent sample set from a completely different health care institution (Duke).

#### Setting 3: Consecutive Outpatient Data

Because of the criteria for usability of the clinical scoring systems for computing pretest probability, we consecutively selected 100 outpatient data from SHC and 101 from Duke and created another hold-out cohort separated from the 2 data sets. We used the models that were trained using SHC patients (setting 1) and tested it on outpatient data separately to judge the model performance of the outpatient cases. In parallel to machine learning models, following the standard procedure for retrospective scoring,^21,22,23,24,25,26^ we scored the 3 pretest clinical scorings—Wells, PERC, and rGeneva—via manual medical record review where an expert (M.P.L.) computed the scoring values based on available EMR data (including notes).

#### Setting 4: Cross-Validation 

To estimate generalizability of the model on a new SHC patient, we performed 10-fold cross-validation on the SHS training data (except holdout test set of 340 CT examinations) (eAppendix and eFigure 2 in the Supplement). A total of 3057 annotated CT examinations were partitioned into 10 equal-sized subsamples in which none of the patients were mixed in training and testing fold. Of the 10 subsamples, a single subsample of approximately 305 CT examinations was retained as the validation data for testing the model, and the remaining 2752 subsamples were used as training data. The cross-validation process was then repeated 10 times, with each of the subsamples used only once as the validation data.

## Results

### Participants

Of the 3214 patients from SHC included in the study, 1704 (53.0%) were women; mean (SD) age was 60.53 (19.43) years. The 240 patients from Duke used for validation included 132 women (55.0%); mean (SD) age was 70.2 (14.2) years. In the samples for clinical scoring system comparisons, the 100 outpatients from SHS included 67 women (67.0%)); mean (SD) age was 57.74 (19.87) years, and the 101 patients from Duke included 59 women (58.4%); mean (SD) age was 73.06 (15.3) years (Table 1).

### Setting 1: Holdout From SHC

**Figure 2.**
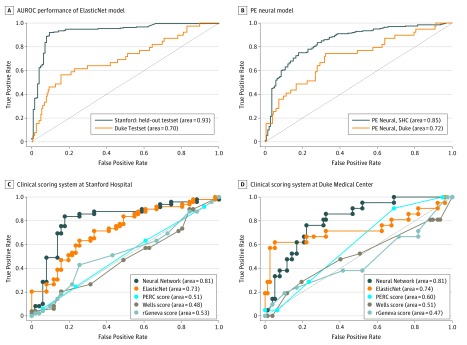
Performance in Setting 1 and 2 A and B, Area under the receiver operating characteristic (AUROC) performance of ElasticNet model (A) and pulmonary embolism (PE) Neural model (B) on holdout internal Stanford hospital and clinics (SHC) and external Duke validation. C and D, Performance in setting 3: comparison with 3 clinical scoring systems on 100 outpatient samples (medical record manually reviewed by experts) seen at SHC (C) and 101 from Duke (D): PERC (Pulmonary Embolism Rule-out Criteria), Wells, rGeneva against our proposed ElasticNet and PE neural model.

**Figure 3.**
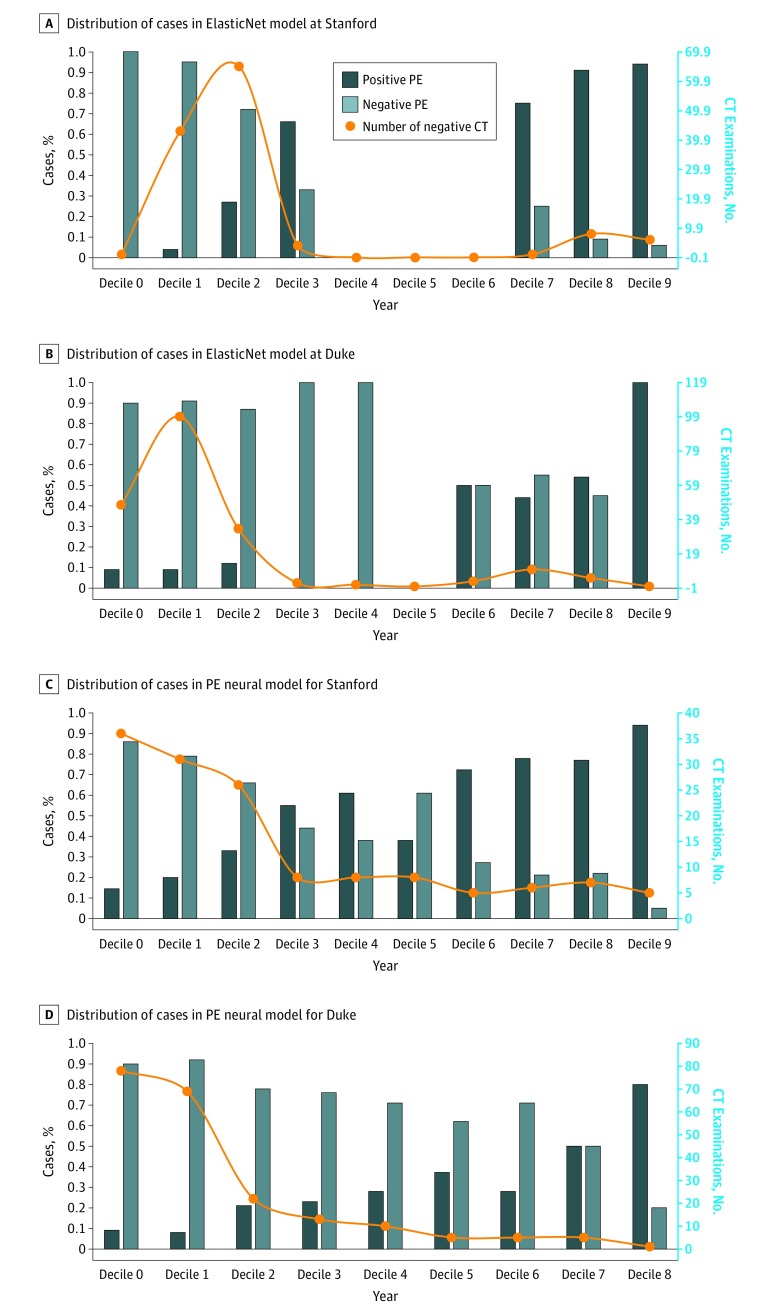
Performance in Setting 1 and 2 Distribution of positive and negative cases in decile of predicted probability value on the primary y-axis and true number of negative computed tomographic (CT) examinations in each decile fitted as a curve on the secondary y-axis: proposed ElasticNet model for the holdout test set from Stanford (A) and Duke (B) and pulmonary embolism (PE) Neural model for the holdout test set from Stanford (C) and Duke (D).

The results are summarized as AUROC in Table 2 and Figure 2; both machine learning models scored high accuracy (PE neural, 0.85; ElasticNet, 0.93), with the ElasticNet model performing significantly better than the PE neural model on the internal data set (*P* = .01). To test the practical usability, we also plotted the distribution of positive and negative cases in decile of predicted probability value along with the number of negative CT examinations (Figure 3). Figure 3 shows that, among 140 negative CT examinations performed at SHC, with a more conservative cutoff level, the ElasticNet model could have redacted 44 of the 140 negative examinations (31.4%) with a cutoff probability lower than 0.2 and only 2 missed PEs. The PE neural model could have redacted 67 of the 140 negative CT examinations (47.9%) with a cutoff probability lower than 0.2 and 4 missed PEs.

**Table 2.** Quantitative Analysis of Model Performance

Variable	SHC Data		Duke Data	
	AUROC	*P* Value	AUROC	*P* Value
**Holdout Testing on Internal and External Data Set**				
ElasticNet model	0.93	.01	0.70	.17
PE neural model	0.85		0.72	
**Comparison With Clinical Scoring Systems on Consecutive Outpatients**				
Machine learning models				
ElasticNet model	0.73	.42	0.74	.01
PE neural model	0.81		0.81	
Clinical scoring				
Wells	0.48	NA	0.51	NA
PERC	0.51		0.60	
rGeneva	0.53		0.47	

Abbreviations: AUROC, area under the receiver operating characteristic curve; Duke, Duke University Medical Center; NA, not applicable; PE, pulmonary embolism; PERC, Pulmonary Embolism Rule-out Criteria; rGeneva, revised Geneva; SHC, Stanford University hospital and clinics.

### Setting 2: External Patient Data From Duke 

As indicated in Table 2 and Figure 2, both machine learning models’ performance dropped on the external Duke data set compared with the internal holdout SHC test set (PE neural, 0.72; ElasticNet, 0.70). However, the AUROC score remained greater than 0.7, and both models performed equally well on the external data (Duke AUROC: ElasticNet, 0.70; PE neural, 0.72; *P* = .17), which suggests that, even if the models have been trained only on the SHC patients, they are generalizable to the Duke patient population. We also plotted the distribution of positive and negative cases in the decile of predicted probability value along with the number of negative CT examinations on the Duke test set (Figure 3). The graph shows that over 244 CT examinations performed at Duke, 205 CT results (84.0%) were negative for PE. With a more conservative cutoff, the ElasticNet model could have redacted 148 of 244 examinations (60.7%) with a cutoff probability of less than 0.2 and 15 missed PEs, and the PE neural model could have redacted 147 of 244 negative CT examinations (60.2%) with a cutoff probability of less than 0.2 and 14 missed PE examinations.

### Setting 3: Comparison With Clinical Scoring Systems 

For head-to-head comparison, the results are shown in Figure 2 as AUROC values for differences in scoring criteria and machine learning model and are summarized in Table 2. The PE neural model performed significantly better (AUROC, 0.81) than all of the other models and criteria on the SHC and Duke holdout outpatient data, including the ElasticNet model on the Duke data (0.74 vs 0.81; *P* = .01). Table 2 also presents the cases with negative results of d-dimer testing from SHC and Duke in which machine learning models resulted in high sensitivity as well as specificity for PE likelihood detection compared with the clinical scorings and d-dimer test.

Overall, the best-performing model achieved an AUROC performance of predicting a positive PE study of 0.90 (95% CI, 0.87-0.91) on intrainstitutional holdout data, with an AUROC of 0.71 (95% CI, 0.69-0.72) on an external data set from Duke; superior AUROC performance and cross-institutional generalization of the model of 0.81 (95% CI, 0.77-0.87) and 0.81 (95% CI, 0.73-0.82), respectively, were noted on holdout outpatient populations from both intrainstitutional and extrainstitutional data. Using conservative cutoff scores (<0.2), we would have avoided 67 of 340 studies at Stanford and 147 of 244 studies at Duke, improving positive CT yield by 78% and 40.2%, respectively.

## Discussion

The purpose of this study was to design and evaluate machine learning models capable of leveraging large volumes of retrospective EMR data to achieve an accurate, patient-specific risk score for PE diagnosis in patients referred for CT imaging. The core contributions of this work include open-source (1) feature engineering pipeline to capture and preserve temporality of EMR data, (2) machine learning models that can use EMR data to predict PE risk in patients referred for CT imaging, and (3) outcome-labeled longitudinal data extracted from EMR data on patients referred for CT imaging for PE.

Systematic attempts to curb unnecessary imaging for PE evaluation have focused on the use of existing predictive PE risk scoring tools, such as Wells or rGeneva, as CDS tools to inform the decision to perform advanced imaging, but in practice have had a disappointing influence on CT imaging yield or use.^27^ Engineering the existing PE risk scoring tools depended on domain experts to designate specific, relevant clinical variables to include in development.^28,29^ Early studies reported AUCROC performance ranging between 0.63 and 0.75; specifically, the rGeneva scoring system, believed by many to be the most widely applicable, consistent, and least subjective of the available predictive models, initially reported an AUROC of 0.74 for both internal and external validation data.^30^ In our outpatient populations, the predictive PE risk scores performance was closer to 0.5, owing to the fact that this population comprised patients referred for CT. Yet, given the low overall yield for positive studies for patients referred for CT imaging for PE, the performance of traditional risk scores, even if they had been systematically applied, may have also been limited by the constraints of rule-based methods.

In contrast to traditional risk scoring tools, newer data-driven machine learning approaches, such as those used in this study, hold the potential to overcome limitations of prior approaches by automatically identifying patterns and dependencies in complex EMR data to make it easier to automatically extract useful information, in this case, for PE prediction.^10,28^ For this work we highlighted 2 techniques—ElasticNet and PE neural model—applied to retrospective structured EMR data and achieved AUROC values of 0.93 and 0.85 on internal holdout data, respectively (performance of other techniques can be found in eTable 4 in the Supplement). The superior results of ElasticNet on internal holdout data and poor generalization to external data may be a reflection of the model’s reliance on dependencies in the training data and overfitting. In contrast, the inherent flexibility of the neural model PERFORM was seen in our results: on external validation of both inpatients and outpatients, the performance loss to the external data set was less pronounced and the performance on the outpatient data achieved an AUROC of 0.81 for both the internal and external outpatient data sets, despite the fact that the population in the external data set was markedly different in terms of factors such as demographics and prevalence of diseases.

We engineered PERFORM to automatically generate an interpretable objective quantification of PE risk, without subjective input, to help inform CT imaging decisions. In our analysis using conservative cutoff scores, we would have avoided 67 of 340 studies at Stanford and 147 of 244 studies at Duke, improving positive CT yield by 78% and 40.2%, respectively; false-negatives, which occurred with this cutoff value, occurred less frequently than current best practice (patients with negative results of d-dimer testing and low probability scores on the traditional risk scores) (eTable 1 in the Supplement). Furthermore, the objective prediction score output may allow earlier treatment for PE in patients with a high positive PE prediction score, which may improve clinical outcomes for those patients as it may allow for rapid, early treatment.^31^ Incorporating PERFORM into clinical workflow would be ideally an intelligent PE imaging CDS rather than an initial triage step; that is, if PE cannot be excluded by initial clinical practice triage with risk scores from tools such as Wells, rGeneva, and CT imaging is then ordered, PERFORM would be triggered to run automatically on the patient’s structured EMR data to provide patient-specific PE risk score information as an informed intervention to improve use at the time of the order (Figure 1A). Encouraging performance on the external Duke data set despite the population difference in terms of variables such as demographics and prevalence of diseases suggests the reproducibility of the PERFORM model.

### Limitations

This study has limitations. This was a retrospective analysis, which carries well-known challenges.^7^ We used CT imaging reports for precise, temporally specific cohort generation to avoid reliance on error-prone claims data that lack information about the exact time of diagnosis.^32,33,34,35^ All patients included in our study underwent CT imaging, even those for whom an age-adjusted d-dimer level would have precluded imaging in most scoring systems. In addition, patients not referred for imaging were excluded because the design of the study was not to displace existing rule-out criteria, but instead to reduce unneeded PE imaging for patients referred for CT imaging for PE.

## Conclusions

The neural network model PERFORM possibly can consider multitudes of patient-specific risk factors and dependencies in retrospective structured EMR data to arrive at an imaging-specific PE likelihood recommendation and may accurately be generalized to new population distributions. The findings of this study suggest that this model may be used as an automated CDS tool to improve use of PE-CT imaging in referred patients.

## Notes

Supplement. **eFigure 1.** Strategy for SHC and Duke Cohort Assembly **eFigure 2.** Performance in Setting 4: 10-Fold Cross Validation Performance of ElasticNet (A) and PE Neural Model (B) on Feature Set of Integrated EMR **eFigure 3.** Population-Level Feature Importance: Importance of Top 22 EMR Feature Based on the Absolute Value of Their Coefficients Learn By the ElasticNet Model **eFigure 4.** Patient-Level Feature Importance: Attention Derived By the PE Neural Model Along With the Predicted Probability of PE Presence **eAppendix.** Detailed Methods and Findings **eTable 1.** D-Dimer Missed Cases From SHC and DUKE Consecutive Out-Patient–D-Dimer Score, Models’ Predicted Probability for PE and Clinical Scorings **eTable 2.** Strategy for Grouping the ICD9 Diagnosis Code for PE Risk Assessment **eTable 3.** Strategy for Grouping the Common Laboratory Tests for PE Risk Assessment **eTable 4.** Comparison Between Linear and Nonlinear Machine Learning Models on the SHC and Duke Hold-Out Test Set **eTable 5.** Grid Search Results for PE Neural Model Hyparameter Tuning on the SHC Validation Set
