# Supplementary material for: Development and Performance of the Pulmonary Embolism Result Forecast Model (PERFORM) for Computed Tomography Clinical Decision Support
Source: JAMA Netw Open. 2019 Aug 7;2(8):e198719. doi: 10.1001/jamanetworkopen.2019.8719 (PMC6686780; doi:10.1001/jamanetworkopen.2019.8719)
Supplement: Supplement. — eFigure 1. Strategy for SHC and Duke Cohort Assembly eFigure 2. Performance in Setting 4: 10-Fold Cross Validation Performance of ElasticNet (A) and PE Neural Model (B) on Feature Set of Integrated EMR eFigure 3. Population-Level Feature Importance: Importance of Top 22 EMR Feature Based on the Absolute Value of Their Coefficients Learn By the ElasticNet Model eFigure 4. Patient-Level Feature Importance: Attention Derived By the PE Neural Model Along With the Predicted Probability of PE Presence eAppendix. Detailed Methods and Findings eTable 1. D-Dimer Missed Cases From SHC and DUKE Consecutive Out-Patient–D-Dimer Score, Models’ Predicted Probability for PE and Clinical Scorings eTable 2. Strategy for Grouping the ICD9 Diagnosis Code for PE Risk Assessment eTable 3. Strategy for Grouping the Common Laboratory Tests for PE Risk Assessment eTable 4. Comparison Between Linear and Nonlinear Machine Learning Models on the SHC and Duke Hold-Out Test Set eTable 5. Grid Search Results for PE Neural Model Hyparameter Tuning on the SHC Validation Set [file jamanetwopen-2-e198719-s001.pdf]

Supplementary Online Content

Banerjee I, Sofela M, Yang J, et al. Development and performance of the Pulmonary Embolism Result Forecast Model (PERFORM) for computed tomography clinical decision support. *JAMA Netw Open.* 2019;2(8):e198719.
doi:10.1001/jamanetworkopen.2019.8719

eFigure 1. Strategy for SHC and Duke Cohort Assembly
eFigure 2. Performance in Setting 4: 10-Fold Cross Validation Performance of ElasticNet (A) and PE Neural Model (B) on Feature Set of Integrated EMR
eFigure 3. Population-Level Feature Importance: Importance of Top 22 EMR Feature Based on the Absolute Value of Their Coefficients Learn By the ElasticNet Model
eFigure 4. Patient-Level Feature Importance: Attention Derived By the PE Neural Model Along With the Predicted Probability of PE Presence
eAppendix. Detailed Methods and Findings
eTable 1. D-Dimer Missed Cases From SHC and DUKE Consecutive Out-Patient – D-Dimer Score, Models' Predicted Probability for PE and Clinical Scorings
eTable 2. Strategy for Grouping the ICD9 Diagnosis Code for PE Risk Assessment
eTable 3. Strategy for Grouping the Common Laboratory Tests for PE Risk Assessment
eTable 4. Comparison Between Linear and Non-Linear Machine Learning Models on the SHC and Duke Hold-Out Test Set
eTable 5. Grid Search Results for PE Neural Model Hyparameter Tuning on the SHC Validation Set

This supplementary material has been provided by the authors to give readers additional information about their work.

© 2019 Banerjee I et al. *JAMA Network Open.*

eFigure 1. Strategy for SHC and Duke Cohort Assembly

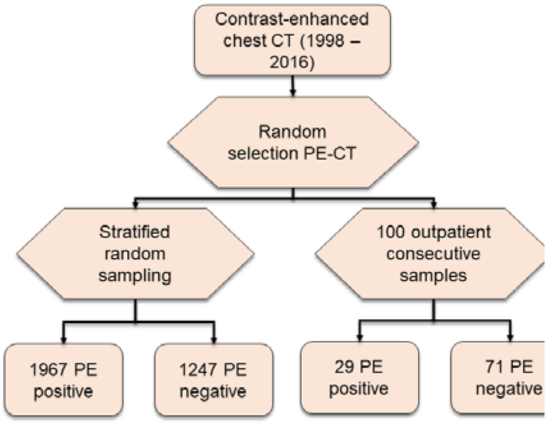

(a) SHC cohort

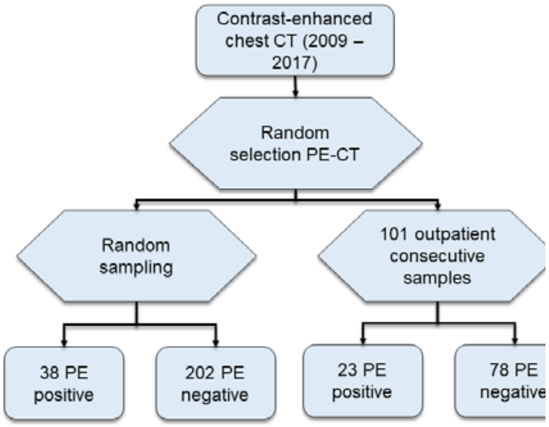

(b) Duke cohort

© 2019 Banerjee I et al. *JAMA Network Open.*

eFigure 2. Performance in Setting 4: 10-Fold Cross Validation Performance of ElasticNet (A) and PE Neural Model (B) on Feature Set of Integrated EMR

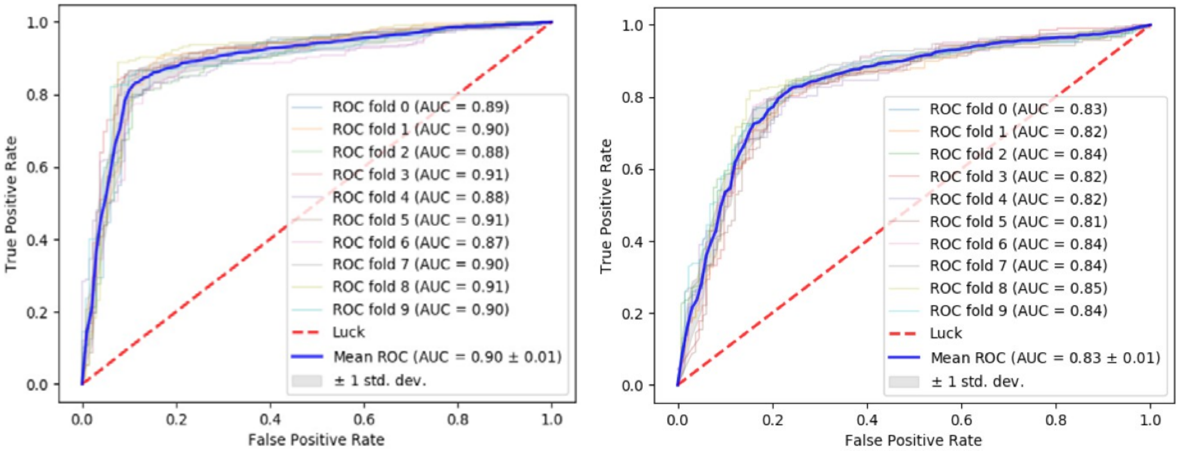

© 2019 Banerjee I et al. *JAMA Network Open.*

eFigure 3. Population-Level Feature Importance: Importance of Top 22 EMR Feature Based on the Absolute Value of Their Coefficients Learn By the ElasticNet Model

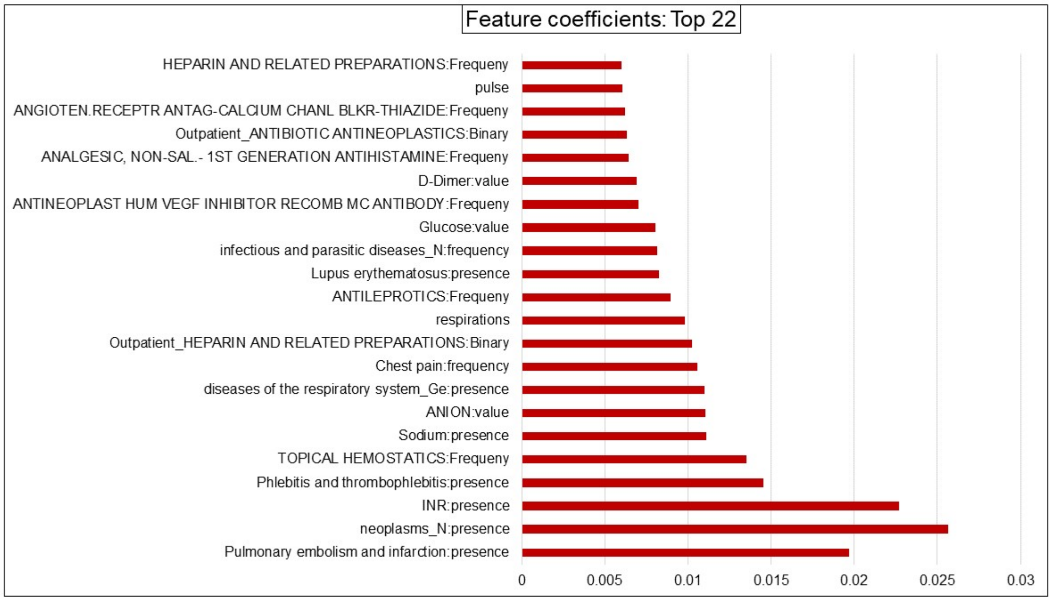

© 2019 Banerjee I et al. *JAMA Network Open.*

eFigure 4. Patient-Level Feature Importance: Attention Derived By the PE Neural Model Along With the Predicted Probability of PE Presence

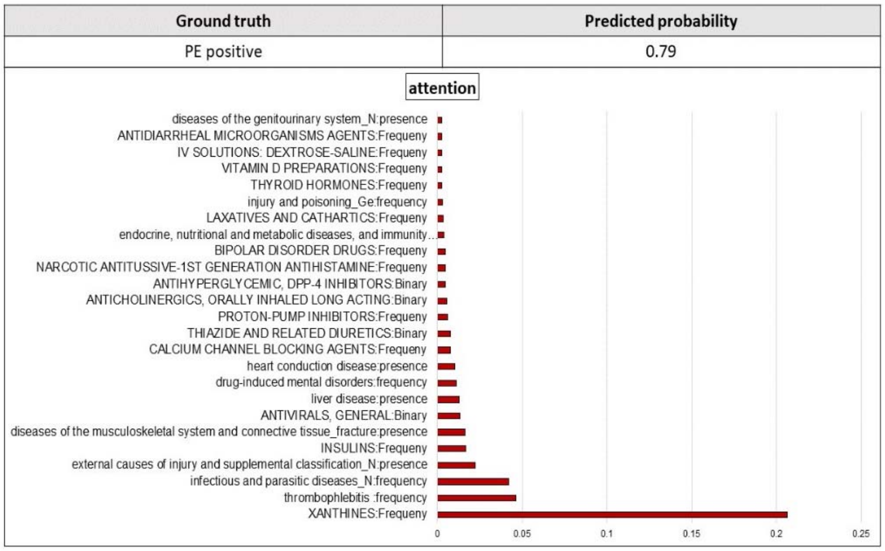

(a) case 1

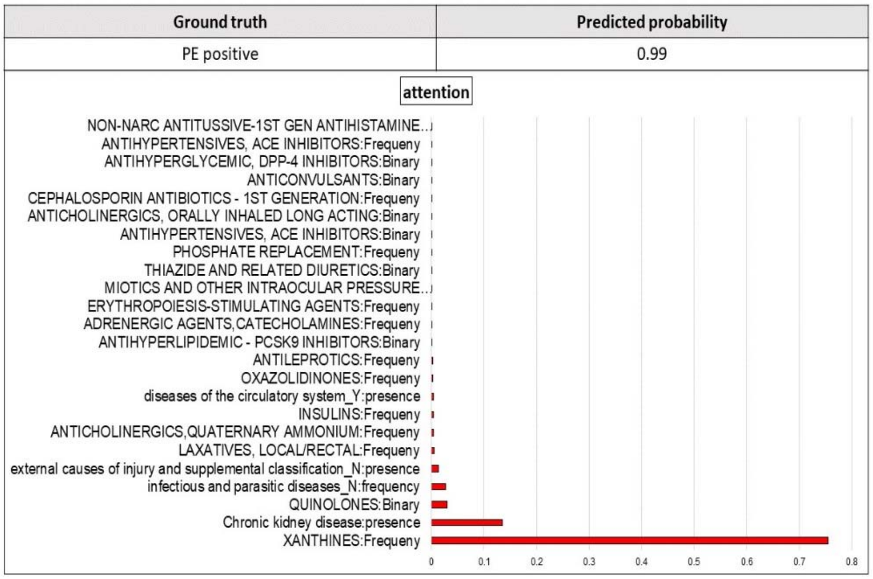

(b) case 2

© 2019 Banerjee I et al. *JAMA Network Open.*

eAppendix. Detailed Methods and Findings

1. Methodology for Temporal Feature engineering

Demographics - As demographics, we considered four static features: gender (male/female), race/ethnicity (white/black/asian/native american/others/unknown), age at time of observation, smoking habit (yes/no) and coded them as categorical variables (age binned into 10 groups). In case of change in smoking status, we only considered the current observation and coded as 'Smoking'/'Non-smoking'.

Vitals - We considered only the primary vital signs of the patient which includes systolic and diastolic blood pressure, height, weight, body mass index (BMI), temperature, respiration rate, pulse oximetry (spO2), and heart rate. For both internal and external datasets, the primary vitals are recorded using the LOINC standard coding system[1]. In order to capture temporality, we measured the sensitivity to change in primary vitals within a 30 day window by computing derivatives of each vital sign along the temporal axis where first value is the normal range of the targeted vital. The derivative of a vital can be represented as $\frac{dY}{dt}$ where $Y = f(X), X = x_N, x_1, x_2, \ldots\ldots x_t$ is a measure of the x vital over time t and x_N is normal range of the targeted vital. Given that majority of the targeted population are adults (with mean age: Stanford 60.53 and Duke 70.2), as normal range x_N we considered vital signs against normal values if prior baseline vitals were not available.

Inpatient and Outpatient medication - The inpatient and outpatient drug formulary and vocabularies were mapped to a 2016 version of RxNorm[2]. Prescription orders were distilled to the Pharmacologic class labels which active moieties that share scientifically documented properties is defined on the basis of any combination of three attributes: Mechanism of Action (MOA), Physiologic Effect (PE), and Chemical Structure (CS), that the FDA has determined to be scientifically valid and clinically meaningful. For drug feature engineering, we considered a 12 month window and identified 641 unique Pharmacologic class of drugs given to the training set SHC patients (inpatient and outpatient). Afterwards, we coded the medication usage as two numeric representations as: (1) presence/absence of the medication which is a binary value that captures if medication from a particular Pharmacologic class given to the patient within the 12 month window; (2) frequency of the medication as a numeric value to captures how many times the particular medication was repeated within 12 months.

Diagnosis code - Diagnosis codes considered were ICD-9 format (codes with less than 1% occurrences in the training set were excluded). In order to limit the learning space, the diagnosis codes were collapsed to the top diagnosis categories using the the International Classification of Diseases, Version 9. Expansion to subcategories was performed with review of ICD9 taxonomy such that in total 141 unique diagnosis groupings (see Supplement Table 1) were generated for each group as a binary variable representing the presence/absence of a particular diagnosis within the 12 month window. For ensuring no data leakage, we dropped all ICD-9 codes recorded from the same encounter (hospitalization and ED visit) as of the CT exam from our analysis.

---

[1] https://www.hl7.org/fhir/observation-vitalsigns.html
[2] https://www.nlm.nih.gov/research/umls/rxnorm/

© 2019 Banerjee I et al. *JAMA Network Open.*

Laboratory tests – All available laboratory tests were categorized into 22 unique test categories (Supplement Table 2). Laboratory tests are coded in binary presence/absence as well as we captured the latest value of each test. Missing lab data is coded as ‘0’ value.

2. Cross-validation performance of the models on SHC patient data

eFigure 1 summarize the 10-fold cross-validation results of the SHC cases. The ElasticNet model performance mean AUC was 0.90+/- 0.01) and the Neural model was (0.83+/- 0.01) with both models showing low variations (+/- 0.01) between the folds which represents high generalizability.

3. D-Dimer missing cases from SHC and Duke consecutive out-patient

© 2019 Banerjee I et al. *JAMA Network Open.*

eTable 1. D-Dimer Missed Cases From SHC and DUKE Consecutive Out-Patient – D-Dimer Score, Models' Predicted Probability for PE and Clinical Scorings

D-Dimer missed cases from SHC and DUKE outpatients							
**Patient**	**D-Dimer value**	**PE Presence**	**ElasticNet**	**Neural network**	**Wells**	**PERC**	**rGeneva**
SHC patients with negative D-Dimer (< 500) - Among 100 patients 29 had D-Dimer and 2 negatives							
1	negative	Yes	0.56	0.16	0	1	3
2	negative	No	0.4	0.015	3	0	3
Duke patients with negative D-Dimer (<500) – Among 101 patients 32 patients had D-Dimer and 4 negatives							
1	negative	Yes	0.76	0.6	1	2	3
2	negative	Yes	0.82	0.84	9	2	8
3	negative	No	0.11	0.056	3	0	3
4	negative	No	0.13	0.03	1.5	3	5

(D-Dimer <500 normal)

4. Models intepretability

ElasticNet model - eFigure 2 shows the trends of the 22 most relevant features for the prediction PE pre-test risk. Looking at the graph we can clearly see that presence of pulmonary embolism and infraction, and neoplasm (cancer) influenced the PE prediction the most. Interesting true value of the D-dimer lab test is also listed as the top features than just the presence of the D-dimer test. Thus we can assume that these features are relevant in order to assess if a new patient has the PE or not.

PE neural model - We used a method called sensitivity analysis for computing the relevance of each EMR feature. Sensitivity analysis takes the partial derivative of the loss function of the trained neural recurrence model with respect to each input feature to derive the importance for the targeted prediction task. eFigure 3 represent results of sensitivity analysis of input for two cases where the importance scores are plotted as bar and predicted probability value and ground truth labels are also shown in the figure.

5. EMR grouping criteria: diagnosis code and laboratory exams

In Section 2.2, we described the proposed feature engineering pipeline that can parse EMR while maintaining the significant temporal properties where the pipeline used pre-defined

© 2019 Banerjee I et al. *JAMA Network Open.*

grouping criteria for diagnosis codes and laboratory test. eTable 2 listed the diagnosis grouping based on ICD9 standard and eTable 3 listed laboratory test grouping which was generated based on discussion with domain experts from both Stanford and Duke sides.

eTable 2. Strategy for Grouping the ICD9 Diagnosis Code for PE Risk Assessment

GROUP	DX SUBGROUP	ICD start	ICD end
CERTAIN CONDITIONS ORIGINATING IN THE PERINATAL PERIOD	MATERNAL CAUSES OF PERINATAL MORBIDITY AND MORTALITY<br>OTHER CONDITIONS ORIGINATING IN THE PERINATAL PERIOD	760<br>764	763.99<br>779.99
COMPLICATIONS OF PREGNANCY, CHILDBIRTH, AND THE PUERPERIUM	COMPLICATIONS MAINLY RELATED TO PREGNANCY<br>COMPLICATIONS OCCURRING MAINLY IN THE COURSE OF LABOR AND DELIVERY	640<br>660	649.99<br>669.99
	COMPLICATIONS OF THE PUERPERIUM<br>ECTOPIC AND MOLAR PREGNANCY	670<br>630	677.99<br>633.99
	NORMAL DELIVERY, AND OTHER INDICATIONS FOR CARE IN PREGNANCY, LABOR, AND DELIVERY<br>OTHER MATERNAL AND FETAL COMPLICATIONS	650<br>678	659.99<br>679.99
	OTHER PREGNANCY WITH ABORTIVE OUTCOME<br>Anencephalus and similar anomalies	634<br>740	639.99<br>740
CONGENITAL ANOMALIES	Anomalies of respiratory system, congenital<br>Bulbus cordis anomalies and anomalies of cardiac septal closure	748<br>745	748<br>745
	Certain congenital musculoskeletal deformities<br>Chromosomal anomalies	754<br>758	754<br>758
	Cleft palate and cleft lip<br>Congenital anomalies of ear, face, and neck	749<br>744	749<br>744
	Congenital anomalies of eye<br>Congenital anomalies of genital organs	743<br>752	743<br>752
	Congenital anomalies of the integument	757	757

© 2019 Banerjee I et al. *JAMA Network Open.*

	Congenital anomalies of urinary system	753	753
	Other and unspecified congenital anomalies<br>Other congenital anomalies of circulatory system	759<br>747	759<br>747
	Other congenital anomalies of digestive system<br>Other congenital anomalies of heart	751<br>746	751<br>746
	Other congenital anomalies of limbs<br>Other congenital anomalies of nervous system	755<br>742	755<br>742
	Other congenital anomalies of upper alimentary tract<br>Other congenital musculoskeletal anomalies	750<br>756	750<br>756
	Spina bifida<br>Acquired hemolytic anemias	741<br>283	741<br>283
DISEASES OF THE BLOOD AND BLOOD-FORMING ORGANS	Aplastic anemia and other bone marrow failure syndromes<br>Coagulation defects	284<br>286	284<br>286
	Diseases of white blood cells<br>Hereditary hemolytic anemias	288<br>282	288<br>282
	Iron deficiency anemias<br>Other and unspecified anemias	280<br>285	280<br>285
	Other deficiency anemias<br>Other diseases of blood and blood-forming organs	281<br>289	281<br>289
	Purpura and other hemorrhagic conditions<br>ACUTE RHEUMATIC FEVER	287<br>390	287<br>392.99
DISEASES OF THE CIRCULATORY SYSTEM	CEREBROVASCULAR DISEASE<br>CHRONIC RHEUMATIC HEART DISEASE	430<br>393	438.99<br>398.99
	DISEASES OF ARTERIES, ARTERIOLES, AND CAPILLARIES<br>DISEASES OF PULMONARY CIRCULATION	440<br>415	449.99<br>417.99
	DISEASES OF VEINS AND LYMPHATICS, AND OTHER DISEASES OF CIRCULATORY SYSTEM<br>HYPERTENSIVE DISEASE	451<br>401	459.99<br>405.99

© 2019 Banerjee I et al. *JAMA Network Open.*

	ISCHEMIC HEART DISEASE<br>OTHER FORMS OF HEART DISEASE	410<br>420	414.99<br>429.99
DISEASES OF THE DIGESTIVE SYSTEM	APPENDICITIS<br>DISEASES OF ESOPHAGUS, STOMACH, AND DUODENUM	540<br>530	543.99<br>539.99
	DISEASES OF ORAL CAVITY, SALIVARY GLANDS, AND JAWS<br>Gastrointestinal mucositis (ulcerative)	520<br>538	529.99<br>538
	HERNIA OF ABDOMINAL CAVITY<br>NONINFECTIOUS ENTERITIS AND COLITIS	550<br>555	553.99<br>558.99
	OTHER DISEASES OF DIGESTIVE SYSTEM<br>OTHER DISEASES OF INTESTINES AND PERITONEUM	570<br>560	579.99<br>569.99
DISEASES OF THE GENITOURINARY SYSTEM	DISEASES OF MALE GENITAL ORGANS<br>DISORDERS OF BREAST	600<br>610	608.99<br>612.99
	INFLAMMATORY DISEASE OF FEMALE PELVIC ORGANS<br>NEPHRITIS, NEPHROTIC SYNDROME, AND NEPHROSIS	614<br>580	616.99<br>589.99
	OTHER DISEASES OF URINARY SYSTEM<br>OTHER DISORDERS OF FEMALE GENITAL TRACT	590<br>617	599.99<br>629.99
DISEASES OF THE MUSCULOSKELETAL SYSTEM AND CONNECTIVE TISSUE	ARTHROPATHIES AND RELATED DISORDERS<br>DORSOPATHIES	710<br>720	719.99<br>724.99
	OSTEOPATHIES, CHONDROPATHIES, AND ACQUIRED MUSCULOSKELETAL DEFORMITIES<br>RHEUMATISM, EXCLUDING THE BACK	730<br>725	739.99<br>729.99
DISEASES OF THE NERVOUS SYSTEM AND SENSE ORGANS	DISEASES OF THE EAR AND MASTOID PROCESS<br>DISORDERS OF THE EYE AND ADNEXA	380<br>360	389.99<br>379.99
	DISORDERS OF THE PERIPHERAL NERVOUS SYSTEM	350<br>330	359.99<br>337.99

© 2019 Banerjee I et al. *JAMA Network Open.*

	HEREDITARY AND DEGENERATIVE DISEASES OF THE CENTRAL NERVOUS SYSTEM		
	INFLAMMATORY DISEASES OF THE CENTRAL NERVOUS SYSTEM<br>ORGANIC SLEEP DISORDERS	320<br>327	326.99<br>327.99
	OTHER DISORDERS OF THE CENTRAL NERVOUS SYSTEM<br>OTHER HEADACHE SYNDROMES	340<br>339	349.99<br>339.99
	PAIN<br>ACUTE RESPIRATORY INFECTIONS	338<br>460	338.99<br>466.99
DISEASES OF THE RESPIRATORY SYSTEM	CHRONIC OBSTRUCTIVE PULMONARY DISEASE AND ALLIED CONDITIONS<br>OTHER DISEASES OF RESPIRATORY SYSTEM	490<br>510	496.99<br>519
	OTHER DISEASES OF THE UPPER RESPIRATORY TRACT<br>PNEUMOCONIOSES AND OTHER LUNG DISEASES DUE TO EXTERNAL AGENTS	470<br>500	478.99<br>508.99
	PNEUMONIA AND INFLUENZA<br>INFECTIONS OF SKIN AND SUBCUTANEOUS TISSUE	480<br>680	488.99<br>686.99
DISEASES OF THE SKIN AND SUBCUTANEOUS TISSUE	OTHER DISEASES OF SKIN AND SUBCUTANEOUS TISSUE<br>OTHER INFLAMMATORY CONDITIONS OF SKIN AND SUBCUTANEOUS TISSUE	700<br>690	709.99<br>698.99
ENDOCRINE, NUTRITIONAL AND METABOLIC DISEASES, AND IMMUNITY DISORDERS	DISEASES OF OTHER ENDOCRINE GLANDS<br>DISORDERS OF THYROID GLAND	249<br>240	259.99<br>246.99
	NUTRITIONAL DEFICIENCIES<br>OTHER METABOLIC AND IMMUNITY DISORDERS	260<br>270	269.99<br>279.99
INFECTIOUS AND PARASITIC DISEASES	ARTHROPOD-BORNE VIRAL DISEASES<br>HELMINTHIASES	60<br>120	66.99<br>129.99

© 2019 Banerjee I et al. *JAMA Network Open.*

	HUMAN IMMUNODEFICIENCY VIRUS [HIV] INFECTION<br>INTESTINAL INFECTIOUS DISEASES	42<br>1	42.99<br>9.99
	LATE EFFECTS OF INFECTIOUS AND PARASITIC DISEASES<br>MYCOSES	137<br>110	139.99<br>118.99
	OTHER BACTERIAL DISEASES<br>OTHER DISEASES DUE TO VIRUSES AND CHLAMYDIAE	30<br>70	41.99<br>79.99
	OTHER INFECTIOUS AND PARASITIC DISEASES<br>OTHER SPIROCHETAL DISEASES	130<br>100	136.99<br>104.99
	POLIOMYELITIS AND OTHER NON-ARTHROPOD-BORNE VIRAL DISEASES AND PRION DISEASES OF CENTRAL NERVOUS SYSTEM<br>RICKETTSIOSES AND OTHER ARTHROPOD-BORNE DISEASES	45<br>80	49.99<br>88.99
	SYPHILIS AND OTHER VENEREAL DISEASES<br>TUBERCULOSIS	90<br>10	99.99<br>18.99
	VIRAL DISEASES GENERALLY ACCOMPANIED BY EXANTHEM<br>ZOONOTIC BACTERIAL DISEASES	50<br>20	59.99<br>27.99
INJURY AND POISONING	BURNS<br>CERTAIN TRAUMATIC COMPLICATIONS AND UNSPECIFIED INJURIES	940<br>958	949.99<br>959.99
	COMPLICATIONS OF SURGICAL AND MEDICAL CARE, NOT ELSEWHERE CLASSIFIED<br>CONTUSION WITH INTACT SKIN SURFACE	996<br>920	999.99<br>924.99
	CRUSHING INJURY<br>DISLOCATION	925<br>830	929.99<br>839.99
	EFFECTS OF FOREIGN BODY ENTERING THROUGH ORIFICE<br>FRACTURES	930<br>800	939.99<br>829
	INJURY TO BLOOD VESSELS<br>INJURY TO NERVES AND SPINAL CORD	900<br>950	904.99<br>957.99

© 2019 Banerjee I et al. *JAMA Network Open.*

	INTERNAL INJURY OF THORAX, ABDOMEN, AND PELVIS	860	869.99
	INTRACRANIAL INJURY, EXCLUDING THOSE WITH SKULL FRACTURE	850	854.99
	LATE EFFECTS OF INJURIES, POISONINGS, TOXIC EFFECTS, AND OTHER EXTERNAL CAUSES	905	909.99
	OPEN WOUNDS	870	897.99
	OTHER AND UNSPECIFIED EFFECTS OF EXTERNAL CAUSES	990	995.99
	POISONING BY DRUGS, MEDICINAL AND BIOLOGICAL SUBSTANCES	960	979.99
	SPRAINS AND STRAINS OF JOINTS AND ADJACENT MUSCLES	840	848.99
	SUPERFICIAL INJURY	910	919.99
	TOXIC EFFECTS OF SUBSTANCES CHIEFLY NONMEDICINAL AS TO SOURCE	980	989.99
	INTELLECTUAL DISABILITIES	317	319.99
MENTAL, BEHAVIORAL AND NEURODEVELOPMENTAL DISORDERS	NEUROTIC DISORDERS, PERSONALITY DISORDERS, AND OTHER NONPSYCHOTIC MENTAL DISORDERS	300	316.99
	PSYCHOSES	290	299.99
NEOPLASMS	NEOPLASMS	140	239.99
	ILL-DEFINED AND UNKNOWN CAUSES OF MORBIDITY AND MORTALITY	797	799.99
SYMPTOMS, SIGNS, AND ILL-DEFINED CONDITIONS	NONSPECIFIC ABNORMAL FINDINGS	790	796.99
	General symptoms	780	780
	Other symptoms involving abdomen and pelvis	789	789
	Symptoms concerning nutrition, metabolism, and development	783	783
	Symptoms involving cardiovascular system	785	785
	Symptoms involving digestive system	787	787
	Symptoms involving head and neck	784	784
	Symptoms involving nervous and musculoskeletal systems	781	781

	Symptoms involving respiratory system and other chest symptoms<br>Symptoms involving skin and other integumentary tissue	786<br>782	786<br>782
SUPPLEMENTARY CLASSIFICATION OF EXTERNAL CAUSES OF INJURY AND POISONING	Symptoms involving urinary system<br>SUPPLEMENTARY CLASSIFICATION OF EXTERNAL CAUSES OF INJURY AND POISONING	788<br>E000	788<br>E999.99
SUPPLEMENTARY CLASSIFICATION OF FACTORS INFLUENCING HEALTH STATUS AND CONTACT WITH HEALTH SERVICES	SUPPLEMENTARY CLASSIFICATION OF FACTORS INFLUENCING HEALTH STATUS AND CONTACT WITH HEALTH SERVICES	V01	V91.99

© 2019 Banerjee I et al. *JAMA Network Open.*

eTable 3. Strategy for Grouping the Common Laboratory Tests for PE Risk Assessment

NAME	GROUP
Albumin	Albumin
Albumin (Serum/Plasma)	
Albumin, Ser/Plas	
Albumin, Serum	
Albumin, Serum/Plas	
ALK P'TASE Total, Serum (Manual Entry) See EMR for details	ALK
Alk P'TASE, Total	
Alk P'TASE, Total, Ser/Plas	
Alkaline Phosphatase	
Alkaline Phosphatase (Serum/Plasma)	
Alkaline Phosphatase Bone	
Alkaline Phosphatase Iso	
Alkaline Phosphatase Total	
Alkaline Phosphatase, Total, Ser/Plas	
ALT	AST
ALT (Manual Entry) See EMR for details	
ALT (SGPT) OSL	
ALT (SGPT), Ser/Plas	
Anion Gap	ANION
ANION GAP	
Anion Gap (Serum/Plasma)	
Anion Gap, ISTAT	
AST	AST
AST (Manual Entry) See EMR for details	
AST (SGOT), Ser/Plas	
BUN	BUN
BUN (Manual Entry) See EMR for details	
BUN, Arterial	
BUN, ISTAT	
BUN, Peripheral	
BUN, Ser/Plas	
BUN, Venous	
BUN/ Creatinine Ratio	BUN/CREATININE
Bun/Creat Ratio	
BUN/Creatinine	
Bun/Creatinine Ratio	
BUN/Creatinine Ratio OSL	
Calcium	Calcium

© 2019 Banerjee I et al. *JAMA Network Open.*

Calcium (Serum/Plasma)	
Creatinine	Creatinine
Creatinine (Serum)	
Creatinine (Serum/Plasma)	
Creatinine, Fluid	
Creatinine, ISTAT	
Creatinine, SER	
Creatinine, Ser/Plas	
Creatinine, Serum	
Creatinine,ISTAT	
D-Dimer	D-Dimer
D-DIMER (MANUAL ENTRY) See EMR for details	
D-Dimer (Plasma/Whole Blood)	
D-Dimer OSL	
D-Dimer, ELISA	
D-Dimer, Elisa	
D-Dimer, Quantitative	
Glucose	Glucose
GLUCOSE	
Glucose - 1 hour	
Glucose - 2 hour	
Glucose - 3 hour	
Glucose - Fasting	
Glucose (Serum/Plasma)	
Glucose (Whole Blood)	
Glucose ,GDM Screen (Serum/Plasma)	
Glucose Non Fasting OSL	
Glucose, ISTAT	
Glucose, Nonfasting	
Glucose, Non-fasting	
Glucose, Nonfasting (Serum/Plasma)	
Glucose, Plasma	
Glucose, Ser/Plas	
Glucose, Serum	
Glucose, WB	
Glucose, Whole Blood	
Glucose,ISTAT	
Hemoglobin	Hemoglobin
Hemoglobin (calc mv), ISTAT	
Hemoglobin (calc), ISTAT	
Hemoglobin (circ), ISTAT	

© 2019 Banerjee I et al. *JAMA Network Open.*

Hemoglobin (HGB)	
Hemoglobin (Manual Entry) See EMR for details	
Hemoglobin (PBG)	
Hemoglobin (xcalc), ISTAT	
Hemoglobin, Plasma	
Hemoglobin A1c	A1C
Hemoglobin A1C	
Hemoglobin A1c (Manual Entry) See EMR for details	
Hemoglobin A1c (Whole Blood)	
Hemoglobin A1c (Whole Blood), POC	
Hemoglobin A1C, POC	
HgB	HgB
Hgb (calc mv), ISTAT	
Hgb (CIRC), ISTAT	
Hgb (circ), ISTAT	
HGB (Manual Entry) See EMR for details	
Hgb (post-oxy calc), ISTAT	
Hgb (xcalc), ISTAT	
INR	INR
INR (Manual Entry) See EMR for details	
INR (Manual)	
INR OSL	
INR, ACLTOP Method	
INR, Fingerstick	
INR, Fingerstick {Menlo}	
INR, ISTAT	
INR, Manual	
INR,POCT	
Lactate (circ), ISTAT	Lactate
Lactate (mv), ISTAT	
Lactate (post-oxy), ISTAT	
Lactate Dehyd(LD), S	
Lactate Dehydrogenase (LDH)	
Lactate Dehydrogenase, Total (Serum/Plasma)	
Lactate(mv), ISTAT	
Lactate(x), ISTAT	
Lactate, ISTAT	
Lactate, Whole Bld	
Lactic Acid	
Lactic Acid (Plasma)	
Lactic Acid OSL	

© 2019 Banerjee I et al. *JAMA Network Open.*

Platelet	Platelet
Platelet count	
Platelet Count	
Platelet Count (Manual Entry) See EMR for details	
Platelet Count (PLT)	
Platelet Ct, Manual	
Platelets	
Potassium	Potassium
Potassium (circ), ISTAT	
Potassium (CIRC), ISTAT	
Potassium (Whole Blood)	
Prothrombin Time	PTT
PROTHROMBIN TIME	
Prothrombin Time (Manual Entry) See EMR for details	
Prothrombin Time, Manual	
PTT	
PTT (Manual)	
Sodium	Sodium
Sodium (circ), ISTAT	
Sodium (CIRC), ISTAT	
Sodium (Manual Entry) See EMR for details	
Sodium (Serum/Plasma)	
Sodium (Whole Blood)	
Sodium (x), ISTAT	
Total Alkaline Phosphatase	AST
Total Alkaline Phosphatase	ALK
Total bile acids	Bilirubin
Total Bilirubin	
Total Bilirubin (Manual Entry) See EMR for details	
Total Bilirubin, Ser/Plas	
Urea Nitrogen (Bun)	BUN
Urea Nitrogen (Serum/Plasma)	
Urea Nitrogen, Ser/Plas	
Urea Nitrogen,Ser/Plas	
Urea Nitrogen/Creatinine (Serum/Plasma)	
WBC	WBC
WBC (Manual Entry) See EMR for details	
WBC count	
WBC Count	
White Blood Cell Count	
White Blood Cells (WBC)	

© 2019 Banerjee I et al. *JAMA Network Open.*

6. Comparison between multiple machine learning models

We experimented with multiple linear and non-linear machine learning models using the same temporal feature vector and reported the performance as AUROC and Negative Predictive Value (NPV) in eTable 4. In the manuscript, we only described the ElasticNet model which resulted the superior performance in terms of AUROC and NPV on both SHC and Duke hold-out test set.

© 2019 Banerjee I et al. *JAMA Network Open.*

eTable 4. Comparison Between Linear and Non-Linear Machine Learning Models on the SHC and Duke Hold-Out Test Set

	**AUROC on SHC data**	**NPV**	**AUROC on Duke data (external testset)**	**NPV**
Hold-out testing on the internal SHC dataset and external Duke dataset (inpatient and outpatient)				
ElasticNet model	0.93	0.81	0.7	0.89
Logistic Regression	0.88	0.79	0.69	0.907
RandomForest	0.9	0.77	0.71	0.9
AdaBoost	0.88	0.79	0.69	0.9

7. Grid search for hyperparameters tuning

For choosing the optimal hyper-parameters for the PE neural model, we applied grid-search on 10% training data as validation set and optimized the validation accuracy. The top 50 hyperparameter settings with training (acc) and validation (val acc) accuracy is summarized in eTable 5.

© 2019 Banerjee I et al. *JAMA Network Open.*

eTable 5. Grid Search Results for PE Neural Model Hyparameter Tuning on the SHC Validation Set

epochs	val loss	loss	acc	val acc	losses	activation	batch size	optimizer	dropout	first neuron	epochs	shape	learning rate	hidden layers
200	0.14	0.05	0.95	**0.85**	mean squared error	<elu>	50	<class ’Adam’>	0.4	200	200	brick	0.0505	2
200	0.14	0.04	0.97	0.85	mean squared error	<elu>	50	<class ’RMSprop’>	0.3	200	200	funnel	0.0901	2
100	0.15	0.04	0.96	0.85	mean squared error	<elu>	50	<class ’Nadam’>	0.4	200	100	brick	0.0703	1
150	0.14	0.07	0.93	0.85	mean squared error	<elu>	50	<class ’Adam’>	0.4	200	150	funnel	0.0208	2
150	0.15	0.04	0.96	0.85	mean squared error	<relu>	100	<class ’RMSprop’>	0.4	200	150	brick	0.0802	2
200	0.15	0.02	0.98	0.85	mean squared error	<relu>	50	<class ’RMSprop’>	0.3	200	200	funnel	0.0604	2
100	0.15	0.03	0.98	0.85	mean squared error	<relu>	50	<class ’Adam’>	0.2	150	100	funnel	0.0802	2
200	0.15	0.04	0.95	0.85	mean squared error	<relu>	100	<class ’Adam’>	0.4	200	200	funnel	0.0406	2

© 2019 Banerjee I et al. *JAMA Network Open.*

100	0.14	0.05	0.95	0.85	mean squared error	<relu>	50	<class ’Adam’>	0.4	150	100	brick	0.0505	1
100	0.14	0.05	0.95	0.85	mean squared error	<elu>	50	<class ’RMSprop’>	0.4	200	100	funnel	0.0901	1
150	0.14	0.03	0.97	0.85	mean squared error	<relu>	50	<class ’RMSprop’>	0.3	200	150	funnel	0.0604	2
100	0.15	0.04	0.96	0.84	mean squared error	<elu>	50	<class ’Nadam’>	0.3	200	100	brick	0.0703	2
200	0.15	0.05	0.95	0.84	mean squared error	<elu>	50	<class ’RMSprop’>	0.2	200	200	brick	0.0208	1
200	0.50	0.06	0.98	0.84	binary crossentropy	<relu>	50	<class ’Adam’>	0.2	100	200	brick	0.0505	2
100	0.48	0.17	0.95	0.84	binary crossentropy	<elu>	50	<class ’RMSprop’>	0.4	200	100	funnel	0.0901	1
100	0.15	0.05	0.95	0.84	mean squared error	<elu>	100	<class ’Nadam’>	0.4	200	100	brick	0.0505	1
150	0.49	0.13	0.96	0.84	binary crossentropy	<relu>	50	<class ’RMSprop’>	0.4	200	150	brick	0.0505	1
150	0.16	0.03	0.96	0.84	mean squared error	<relu>	50	<class ’RMSprop’>	0.3	200	150	brick	0.0505	2

© 2019 Banerjee I et al. *JAMA Network Open.*

200	0.15	0.05	0.95	0.84	mean squared error	<elu>	50	<class ’RMSprop’>	0.4	100	200	brick	0.0505	1
150	0.15	0.06	0.94	0.84	mean squared error	<elu>	100	<class ’RMSprop’>	0.3	150	150	brick	0.0406	2
50	0.15	0.11	0.88	0.84	mean squared error	<elu>	50	<class ’RMSprop’>	0.4	150	50	funnel	0.0406	2
200	0.51	0.07	0.98	0.84	binary crossentropy	<elu>	50	<class ’Nadam’>	0.4	200	200	brick	0.0802	1
100	0.15	0.02	0.98	0.84	mean squared error	<relu>	50	<class ’Nadam’>	0.2	150	100	funnel	0.0802	2
200	0.15	0.03	0.96	0.84	mean squared error	<relu>	50	<class ’RMSprop’>	0.4	200	200	brick	0.0505	0
200	0.15	0.02	0.98	0.84	mean squared error	<relu>	50	<class ’Adam’>	0.2	100	200	funnel	0.0901	2
50	0.15	0.04	0.96	0.84	mean squared error	<elu>	50	<class ’Nadam’>	0.2	200	50	brick	0.0802	1
150	0.15	0.03	0.97	0.85	mean squared error	<relu>	100	<class ’Nadam’>	0.4	200	150	funnel	0.0406	1

© 2019 Banerjee I et al. *JAMA Network Open.*

200	0.15	0.03	0.97	0.84	mean squared error	<elu>	200	<class ’Nadam’>	0.3	200	200	funnel	0.0802	1
50	0.15	0.08	0.92	0.84	mean squared error	<elu>	50	<class ’RMSprop’>	0.3	100	50	funnel	0.0703	2
100	0.15	0.04	0.96	0.84	mean squared error	<elu>	50	<class ’RMSprop’>	0.1	100	100	brick	0.0802	2
100	0.15	0.03	0.97	0.84	mean squared error	<elu>	50	<class ’Nadam’>	0.3	200	100	funnel	0.0703	2
100	0.15	0.04	0.96	0.84	mean squared error	<elu>	50	<class ’Adam’>	0.3	200	100	funnel	0.0703	1
100	0.16	0.03	0.97	0.84	mean squared error	<relu>	50	<class ’Adam’>	0.2	200	100	funnel	0.0604	2
200	0.15	0.04	0.96	0.84	mean squared error	<relu>	50	<class ’Adam’>	0.4	150	200	brick	0.0406	2
150	0.15	0.02	0.98	0.84	mean squared error	<relu>	100	<class ’Nadam’>	0.2	100	150	funnel	0.0802	1
150	0.15	0.03	0.96	0.84	mean squared error	<relu>	100	<class ’Adam’>	0.4	200	150	brick	0.0703	1

© 2019 Banerjee I et al. *JAMA Network Open.*

150	0.15	0.04	0.96	0.84	mean squared error	<elu>	50	<class 'RMSprop'>	0.2	150	150	brick	0.0703	2
100	0.15	0.02	0.98	0.84	mean squared error	<relu>	50	<class 'Nadam'>	0.2	200	100	funnel	0.0802	1
100	0.49	0.24	0.92	0.84	binary crossentropy	<relu>	50	<class 'RMSprop'>	0.4	200	100	funnel	0.0406	2
100	0.15	0.04	0.96	0.84	mean squared error	<elu>	50	<class 'Nadam'>	0.1	200	100	brick	0.0109	2
50	0.16	0.04	0.96	0.84	mean squared error	<relu>	100	<class 'Nadam'>	0.3	150	50	funnel	0.0802	2
50	0.15	0.05	0.95	0.84	mean squared error	<elu>	50	<class 'Nadam'>	0.3	200	50	funnel	0.0505	2
150	0.15	0.03	0.97	0.84	mean squared error	<elu>	50	<class 'Nadam'>	0.3	200	150	brick	0.0901	1
200	0.16	0.02	0.98	0.84	mean squared error	<relu>	100	<class 'Nadam'>	0.3	150	200	brick	0.0604	0
150	0.15	0.03	0.97	0.84	mean squared error	<elu>	100	<class 'Nadam'>	0.2	150	150	brick	0.0802	1

© 2019 Banerjee I et al. *JAMA Network Open.*

150	0.15	0.02	0.98	0.84	mean squared error	<relu>	50	<class ’RMSprop’>	0.3	200	150	funnel	0.0802	1
50	0.15	0.10	0.89	0.84	mean squared error	<elu>	50	<class ’RMSprop’>	0.4	150	50	funnel	0.0505	2
200	0.50	0.05	0.98	0.84	binary crossentropy	<relu>	50	<class ’RMSprop’>	0.2	200	200	funnel	0.0505	1
100	0.15	0.04	0.96	0.84	mean squared error	<relu>	50	<class ’RMSprop’>	0.2	150	100	funnel	0.0406	2
100	0.49	0.21	0.94	0.84	binary crossentropy	<relu>	50	<class ’RMSprop’>	0.4	100	100	brick	0.0802	2

© 2019 Banerjee I et al. *JAMA Network Open.*
